# Analysis of Adult Neural Retina Extracellular Vesicle Release, RNA Transport and Proteomic Cargo

**DOI:** 10.1167/iovs.61.2.30

**Published:** 2020-02-21

**Authors:** Jason Mighty, Jing Zhou, Alberto Benito-Martin, Sami Sauma, Samer Hanna, Onyekwere Onwumere, Cui Shi, Martin Muntzel, Moira Sauane, Michael Young, Henrik Molina, Dianne Cox, Stephen Redenti

**Affiliations:** 1 Department of Biological Sciences, Lehman College, City University of New York, Bronx, New York, United States; 2 Biology Doctoral Program, The Graduate School and University Center, City University of New York, New York, United States; 3 Children's Cancer and Blood Foundation Laboratories, Departments of Pediatrics, and Cell and Developmental Biology, Drukier Institute for Children's Health, Meyer Cancer Center, Weill Cornell Medical College, New York, United States; 4 Department of Anatomy and Structural Biology, Albert Einstein College of Medicine, Bronx, New York, United States; 5 Biochemistry Doctoral Program, The Graduate School and University Center, City University of New York, New York, United States; 6 The Schepens Eye Research Institute, Massachusetts Eye and Ear, Harvard Medical School, Boston, Massachusetts, United States; 7 Proteomics Resource Center, The Rockefeller University, New York, United States

**Keywords:** extracellular vesicles, retina, exosomes, proteomic, cell-to-cell transport

## Abstract

**Purpose:**

Extracellular vesicles (EVs) contain RNA and protein cargo reflective of the genotype and phenotype of the releasing cell of origin. Adult neural retina EV release, RNA transfer, and proteomic cargo are the focus of this study.

**Methods:**

Adult wild-type mouse retinae were cultured and released EV diameters and concentrations quantified using Nanosight. Immunogold transmission electron microscopy (TEM) was used to image EV ultrastructure and marker protein localization. Quantitative real-time polymerase chain reaction (qRT-PCR) was used to analyze retinal cell transcripts present in EVs. Super-resolution microscopy was used to image fluorescent (green) RNA and (red) lipid membrane labeled EVs, released by adult retina, and internalized by isolated retinal cells. Mass spectrometry was used to characterize the proteomes of adult retina and EVs.

**Results:**

Adult neural retina released EVs at a rate of 1.42 +/− 0.08 × 10^8^/mL over 5 days, with diameters ranging from 30 to 910 nm. The canonical EV markers CD63 and Tsg101 localized to retinal EVs. Adult retinal and neuronal mRNA species present in both retina and EVs included rhodopsin and the neuronal nuclei marker NeuN. Fluorescently labeled RNA in retinal cells was enclosed in EVs, transported to, and uptaken by co-cultured adult retinal cells. Proteomic analysis revealed 1696 protein species detected only in retinal cells, 957 species shared between retina and EVs, and 82 detected only in EVs.

**Conclusions:**

The adult neural retina constitutively releases EVs with molecular cargo capable of intercellular transport and predicted involvement in biological processes including retinal physiology, mRNA processing, and transcription regulation within the retinal microenvironment.

The mammalian retina releases a range of extracellular soluble factors active in development, neurophysiology, disease and neuroprotection.^1^^–^^3^ Established soluble factors present during retinal development that are involved in cell division and fate determination include basic fibroblast growth factor (bFGF), transforming growth factor alpha (TGFα), and leukemia inhibitory factor (LIF). In the adult retina, a number of neurotrophic factors are released from Muller glia, which include ciliary neurotrophic factor (CNTF) and brain-derived neurotrophic factor (BDNF).^1^^–^^3^ Adult amacrine and ganglion cells express Wingless-INT (WNT) and the ligand has predicted involvement in retinal homeostasis.^4^^,^^5^ In this work, we begin to define a novel form of diffusible retinal signaling, by characterizing the release kinetics, ultrastructure, and molecular content of extracellular vesicles (EVs) released in the adult retinal microenvironment.

EVs are a family of lipid enclosed, cell-derived micro- and nano-vesicles that facilitate paracrine and autocrine-type signaling via transfer of organelles, DNA, RNA, and protein species.^6^^,^^7^ EVs encapsulate molecular factors that reflect the cell of origin genotype and phenotype. They have diameters ranging from 30 nm to 1 µm and are released from all cell types, including embryonic stem cells, malignant cells, neurons, and glia.^7^^,^^8^ EVs may act locally or circulate systemically, and have been extracted from tears, blood, urine, saliva, and cerebral spinal fluid.^9^ Because of their unique cell of origin specific molecular cargo and systemic bioavailability, EVs are being investigated as mediators of health and disease, as well as prognostic markers of neurodegeneration, cancer, cardiovascular, and immune system diseases.^10^

A subtype of EVs is exosomes, formed through the endosomal-sorting complex required for transport (ESCRT) with average diameters of 30 to 130 nm.^11^ Exosome genesis occurs through three primary processes: 1) initiation or early endosome formation, 2) multivesicular body (MVB) formation, and 3) exosome secretion. The initial stage of exosome formation involves invagination of plasma membrane where vesicles are targeted to early endosomes. Within the ESCRT system, endosomes and intraluminal vesicles are formed at MVBs and are either transported to lysosomes for degradation or exocytosed as exosomes through the plasma membrane via calcium-dependent mechanisms.^12^ Canonical markers for exosomes include tetraspanins and plasma membrane proteins, such as tetraspanins (CD9 and CD63), Alix, and TSG101.^13^

An additional subpopulation of EVs, distinct from exosomes in genesis and content are microvesicles, with diameters ranging from 100 nm to 1 µm.^14^ Microvesicle genesis results from plasma membrane invagination, via transferase interaction with phosphatidylserine proteins, leading to vesicle translocation to the outer membrane leaflet of the cell surface.^15^ The contraction of actin-myosin filaments within the cell cytoskeleton completes the budding process, releasing microvesicles into the extracellular space. Established markers for microvesicles include integrins, selectins, and membrane proteins, such as CD40.^16^^,^^17^

The cargo of stem and neural progenitor cell EVs has been associated with pluripotency, developmental signaling, and neuronal differentiation.^18^ EVs secreted from human embryonic stem cells (hESCs) contain factors that induce pluripotency, namely oct-4, nanog, and gata-4.^19^ Similarly, induced pluripotent stem cell (iPSC) EVs encapsulates mRNA of the transcription factors Oct-3/4, Nanog, Klf4, and C-Myc.^20^ Our recent analysis of retinal progenitor cell (RPC) EVs revealed cargo associated with multipotency, including Pax6, Hes1, Ki67, and Nestin. Using a Cre-loxP system, functional transfer of EV molecular cargo was demonstrated between RPC populations.^21^^,^^22^

Adult central nervous system (CNS) EV function includes maintenance of neurogenic niches, neuroprotection, and modulation of synaptic plasticity.^23^^,^^24^ Neuroprotective astrocyte EV cargo includes Hsp/Hsc70, synapsin, and FGF-2.^25^ Oligodendrocyte EVs facilitate neurite outgrowth, reduce inflammation, and transfer mRNA to axons supporting axon structure and metabolic activity.^26^ Depolarization enhanced release of EV cargo is predicted to facilitate adult plasticity and contains Ca^2+^/calmodulin-dependent protein kinase, neuronal adhesion molecule L1, and AMPA receptor subunits.^27^^,^^28^

In this work, we characterize the total EV population released from adult retina, describing release rates, RNA transfer, and proteomic cargo. A constitutive release rate was observed with EVs exhibiting ultrastructure and marker localization consistent with exosome and microvesicle characterization. A subset of transcripts expressed in adult retina was also detected in EV mRNA cargo. EV encapsulation and transfer of mRNA between adult retinal cells was visualized, revealing EV uptake and cytoplasmic localization. Comparative proteomic profiles of retinal tissue and EVs showed 954 shared protein species with 82 detected only in EVs. The predicted functional categories associated with the retinal EV proteome included protein binding, nucleotide binding, transferase activity, DNA, and RNA binding. Adult retinal EVs may prove to mediate retinal homeostasis and disease processes, with the potential of serving as prognostic markers for retinal disease.

## Methods

### Retina Isolation and Culture

All experiments were approved by and performed in compliance with the City University of New York, Lehman College Animal Care and Use Committee (IACUC). C57BL/6J mice, aged 3 to 6 months, were euthanized and whole eyes were collected (*n* = 10). The anterior segments were cut away at the ora serrata. The vitreous and retinal pigment epitheliums (RPE) were removed from neural retinas. Neural retinas were cultured in 6-well plates at 37°C in 5 mL of Dulbecco’s Modified Eagle Medium (DMEM), plus 10% exosome free fetal bovine serum (FBS), 1% penicillin-streptomycin (Millipore Sigma) and 0.2% Nystatin (Millipore Sigma). Retina conditioned media and retinal tissues were collected after five days for analysis.

### NanoSight Analysis

EV diameters and concentrations were quantified using the NanoSight NS500 system. Retina conditioned media (*n* = 3) were centrifuged at 300  ×  *g* for 10 minutes at 4°C to pellet cell debris. Supernatants were transferred to ultracentrifuge tubes (Beckman Coulter), and spun at 10,000  ×  *g* for 20 minutes using 60Ti rotor (Beckman Coulter ultracentrifuge) at 4°C. Control nonconditioned medium was processed in triplicate using identical incubation periods and isolation steps. Supernatant was diluted at 1:20 in phosphate-buffered saline (PBS) and 1 mL was used for NanoSight analysis. Nanoparticle Tracking Analysis (NTA) software version 2.3 was used to track movements and diameters of nanoparticles. Results are displayed as frequency sized distribution graphs, showing the number of particles per milliliter. The concentration of released EVs was calculated to determine the average number of EVs along with SDs in conditioned media and in control, nonconditioned media, and was analyzed using Student's *t*-tests.

### EV Isolation

Retina conditioned media were collected from 6-well plates and extracted with Exoquick TC (System Biosciences). Briefly, conditioned media was collected and centrifuged at of 3000 x *g* for 15 minutes to remove cell debris. The supernatant was collected, mixed with Exoquick TC solution, and inverted five times. The solution was then incubated at 4°C for 12 hours followed by centrifugation at 1500 ×*g* for 30 minutes. Post-spin supernatant was aspirated and an EV pellet collected. The pellet was then analyzed for RNA and protein content.

### Retina and EV RNA Isolation

Total RNA was extracted and purified from EV pellets and retinal tissue using the RNeasy Plus Mini Kit (Qiagen), according to the manufacturer’s protocol. Briefly, the EV pellet or retinal tissue was mixed with 350 µL RLT Plus buffer and 10 µL β-Mercaptoethanol and vortexed for 15 seconds. The lysate was transferred to a gDNA eliminator spin column, placed in a 2 mL collection tube, and centrifuged for 30 seconds at 8100 × *g*, the flow-through was mixed with 350 µL of 70% ethanol, transferred to an RNeasy spin column in a 2 mL collection tube, centrifuged for 15 seconds at 8100 ×x *g*, and the flow-through discarded. Next, 700 µL of RW1 buffer was added to the RNeasy mini spin column, centrifuged for 15 seconds at 8100 × *g*, and the flow-through was discarded. Then, 500 µL of RPE buffer was added to the RNeasy spin column and centrifuged for 15 seconds at 8100 × *g* and the flow-through was discarded. Following this, 500 µL of RPE buffer was added to the RNeasy spin column and centrifuged for 2 minutes at 8100 × *g*. Last, the RNeasy spin column was placed in a new 1.5 mL collection tube, 50 µL of RNase-free water added, and centrifuged for 1 minute at 8000 x *g* to elute the RNA. RNA was screened for purity and concentration using a Nanodrop-1000 spectrophotometer.

### Western Blot Analysis

Total protein was extracted from EVs and retinal tissues using Pierce IP lysis Buffer (50 µL, Thermo Scientific Fisher) and 100X protease inhibitor. Protein lysates were centrifuged for 15 minutes at 12,000 RPM at 4°C. The supernatants were collected and mixed with 6X sample buffer and heated at 75° C for 15 minutes. Following normalization, 5 µg of each protein sample was added to 4% to 12% gels, loaded into a Mini Gel Tank (Invitrogen), filled with 1X running buffer, and run at 200 V for 45 minutes. The gels were transferred to a nitrocellulose membrane and incubated in Odyssey blocking solution for 45 minutes followed by overnight incubation with primary antibody at 4°C. The membrane was then rinsed 3 times with 1X TBS-T for 5 minutes each followed by incubation with secondary antibody for 45 minutes at 4°C. The membrane was washed 3 times with 1X TBS-T for 5 minutes each, and protein bands analyzed using the Odyssey Clx imaging system.

### Quantitative Real-Time Polymerase Chain Reaction

Total RNA (1 µg) was reverse-transcribed to produce complementary DNA (cDNA) using the New England Biolabs Protoscript AMV First Strand cDNA Synthesis Kit, according to the manufacturer's protocol. Briefly, isolated RNA was mixed with d (T) 23 primer and denatured for 5 minutes at 70° C. The AMV reaction mix buffer, an enzyme mix of reverse transcriptase and murine RNase inhibitor, was added to the sample and incubated at 42°C for 1 hour. The AMV enzyme was inactivated via incubation at 80°C for 5 minutes. The cDNA product was diluted with 30 µL nuclease-free water and analyzed using SYBR Green ER quantitative real-time polymerase chain reaction (qRT-PCR) Supermix. According to the iCycler protocol, 12.5 µL of SYBR GreenER qRT-PCR Supermix, 0.5 µL of forward and reverse primers, and 1 µL of cDNA template were combined, and DEPC water was added to bring the volume to 25 µL. Samples were prepared in biologic triplicates and mixed in a PCR plate followed by amplification and melting curve analysis using a Bio-Rad iCycler. RT-PCR was carried out in biologic triplicates for each primer pair (Table). Student's *t*-test was used to identify significant differences in mRNA, Ct values, between retina and EVs, and *P* values of < 0.05 were considered statistically significant.

**Table.** Primers and Product Sizes for qRT-PCR

Gene	Primer Sequence (5′–3′)	(bp)
Klf14	F: TCT TGG ATT TGG GGT GAG AG	276
	R: GGG ATC ATA GGG GAC CTC AT	
Smoc	F: GGG ACT TCC ACA CGC TAT GT	169
	R: CCT GAA CCA TGT CTG TGG TG	
Igsf8	F: GCA CCG CTG TCT CTA TCT CC	293
	R: GTT GCC CAG GTA CTG CGT AT	
Gprc5b	F: AGT TCA AAC GGT GGA AGG TG	247
	R: TAG TTG GGT GGG TTC TCC TG	
β-actin	F: TTC CAG CCT TCC TTC TTG	
	R: GGA GCC AGA GCA GTA ATC	
GAPDH	F: AAC TTT GGC ATT GTG GAA GG	223
	R: ACA CAT TGG GGG TAG GAA CA	

### Transmission Electron Microscopy 

EVs isolated from retina-conditioned media were fixed in 2.5% glutaraldehyde with 4% paraformaldehyde (EM grade) for 2.5 hours and washed in PBS for 24 hours. They were post-fixed in osmium tetraoxide for 30 minutes, washed with distilled water, and then dehydrated using increasing ethanol concentrations (70%, 85%, 95%, and 100%), each for 10 minutes. Sample dehydration was followed by immersion in propylene for 20 minutes, twice. Samples were infiltrated with a 1:1 mixture of propylene oxide and Spurr's Resin for 1 hour, and then left in 100% Spurr's Resin overnight. Samples were then embedded in beem capsules using fresh Spurr's Resin at 70°C for polymerization. Excess resin was trimmed and 90 nm sections of samples were made using a Leica Ultramicrotome. Sections were placed on 200 mesh copper grids, stained with saturated uranyl acetate in 50% ethanol for 6 minutes, rinsed in water, and stained for 90 seconds in lead citrate. Grids were then rinsed in water, dried on filter paper, and viewed under a Fei Tecnai transmission electron microscope operated at 80 kV. Images were obtained using an AMT camera with AMT digital software.

### Immunoelectron Microscopy

Five µL of re-suspended 2% paraformaldehyde fixed EVs were put on glow-discharged formvar-carbon coated nickel grids. After washing with PBS, the grids were incubated with 50 mM glycine/PBS for 3 minutes. The grids were then blocked for 10 minutes with 1% cold-water fish skin gelatin (Sigma-Aldrich) for surface immunolabeling. Primary antibodies (Tsg101 and CD63) were suspended in blocking solution and applied for 2 hours at room temperature. Controls were prepared identically but in the absence of primary antibodies. After washing with PBS, nanogold-labeled Fab’ anti-rabbit, anti-mouse (Nanoprobes, Yaphank, NY, USA), as well as 5 nm or 10 nm gold conjugated goat anti-mouse antibodies (Ted Pella Inc., Redding, CA, USA) were added to their corresponding antibody incubation buffer and then incubated for 1 hour. The grids were then washed with PBS and fixed in 1% glutaraldehyde for 5 minutes. After thoroughly washing with distilled water, the grids were either placed directly into methylcellulose for 5 nm or 10 nm gold embedding or allowed to continue with silver enhancement of nanogold. For the silver enhancement, the grids were washed with 0.02 M sodium citrate (pH 7.0), and enhancement was performed in the dark using HQ Silver enhancement kit (Nanoprobes) at room temperature for 8 minutes. After washing with distilled water, the grids were contrasted and embedded in a mixture of 3% uranyl acetate and 2% methylcellulose in a ratio of 1:9. Stained grids were examined under a Philips CM-12 electron microscope and photographed with a Gatan (1k × 1k) digital camera. All antibodies were purchased from Abcam, USA, and Anti-Tsg101 (1:200) and CD63 (1:200) were diluted according to manufacturer's instructions. Imaging as described above followed this.

### Retinal RNA Labeling and EV RNA Cargo Transfer Analysis

Using the retinal culture and isolation methods described above, the RNA specific dye SytoRNA Select (Invitrogen) was used on whole adult mouse retinas with minor modifications to the manufacturer's protocol. Briefly, retinas were placed in a 2.5 uM solution of dye and incubated at 37°C in 5% CO2 for 1 hour and then rinsed 3 times with fresh medium. SytoRNA labeled retinas were then incubated in the TRITC fluorescent lipophilic dye PKH26 (Sigma) according to manufacturer's instructions. Briefly, retinas were placed in a 4 × 10^−6^ M dye solution, and incubated at room temperature for 15 minutes on an orbital shaker. One mL of HI-FBS was then used to quench labeling. SytoRNA and PKH26 labeled retinas were washed in fresh culture medium 3X and transferred to 6-well plates containing fresh medium.

### Transwell EV Diffusion Culture

Fresh retinas were collected as described above, lightly dissociated by trituration, and single cells cultured in 12 well glass bottom plates (MatTek Corporation). Next, whole retinas stained with SytoRNA Select (Invitrogen) and PKH26 (Sigma), above, were cultured in transwell inserts with 0.4 µm pore PET membranes suspended above nonlabeled single retina cells and co-cultured for 5 days. Double labeled SytoRNA (green) RNA and PKH26 (red) lipid EVs released by adult retinas, diffused through the transwell membrane pores, and were imaged in contact with and internalized by single cells on the glass-bottom wells.

### Super Resolution Imaging of Retinal Cells Containing Transferred EVs

Following the transwell diffusion of double-labeled EVs described above, single adult retinal neurons from glass-bottom wells containing adherent or internalized EVs were fixed with 4% PFA and mounted with DAPI Prolong Gold mounting medium for imaging. Multichannel structured illumination microscopy (SIM) images were acquired using a Nikon Structured Illumination N-SIM system on an inverted Nikon ECLIPSE Ti-E equipped with a 100 × 1.49 NA objective. Multicolor fluorescence was generated using diode lasers (488, 561, and 647 nm). Z stack images were acquired using the electron-multiplying CCD cameras (Andor iXon3 DU897) with 512 × 512 pixel frame size. Three reconstruction parameters (Illumination Modulation Contrast, High Resolution Noise Suppression, and Out of Focus Blur Suppression) were extensively tested to generate consistent images across experiments, while eliminating abnormal features or artifacts and producing the best Fourier transforms. The acquired images were then processed using Nikon Elements software. The 3D reconstruction was generated using Imaris software (Bitplane).

### Mass Spectrometry

Whole retina lysate and EV samples from mouse retinas were denatured in 8 M urea, reduced with 10 mM DTT, and alkylated with 50 mM iodoacetamide. This was followed by overnight proteolytic digestion with endoproteinase LysC (Wako Chemicals) and then trypsin (Promega) for 6 hours at room temperature. The digestion was quenched with 2% formic acid and the resulting peptide mixtures were desalted using in-house made C18 Empore (3M) StAGE tips.^29^ Samples were dried and resolubilized in 2% acetonitrile and 2% formic acid. Approximately 1 µg of each sample was injected for analysis by reversed phase nano-LC-MS/MS (Ultimate 3000 coupled to a QExactive Plus; Thermo Scientific). After loading onto a C18 trap column (PepMap, 5 µm particles, 100 µm × 2 cm; Thermo Scientific), peptides were separated using a 12 cm × 75 µm C18 column (3 µm particles; Nikkyo Technos Co., Ltd., Tokyo, Japan) at a flow rate of 200 nL/minute, with a gradient increasing from 5% Buffer B (0.1% formic acid in acetonitrile)/95% Buffer A (0.1% formic acid) to 40% Buffer B/60%Buffer A, over 140 minutes. All liquid-chromatography tandem mass spectrometry (LC-MS/MS) experiments were performed in data-dependent mode with lock mass of m/z 445.12003.^29^ Precursor mass spectra were recorded in a 300 to 1400 m/z range at a 70,000 resolution, and fragment ions at a 17,500 resolution (lowest mass: m/z 100). Up to 20 precursors per cycle were selected for fragmentation and dynamic exclusion was set to 60 seconds. Normalized collision energy was set to 27.

### Proteomic Profiling Analysis

Mass spectrometry data were searched against a Uniprot mouse database (July 2014) using MaxQuant (version 1.5.0.30).^23^^,^^24^ Oxidation of methionine and N-terminal protein acetylation were allowed as variable modifications, whereas all cysteines were treated as being carbamidomethylated. Precursor mass tolerance was set at 4.5 ppm, whereas a 20 ppm tolerance was allowed for fragment ions. Two missed cleavages were allowed for specific tryptic search. The “match between runs” option was enabled. False discovery rates at the protein and peptide level were set to 1%. Protein abundances were represented by Label Free Quantitation (LFQ) and intensity-Based Absolute Quantitation (iBAQ).^29^ The iBAQ values were log2(x) transformed and were used to create box plots to depict the distribution and changes in protein expression between two samples.

## Results

### Concentration and Diameters of Retinal EVs

**Figure 1.**
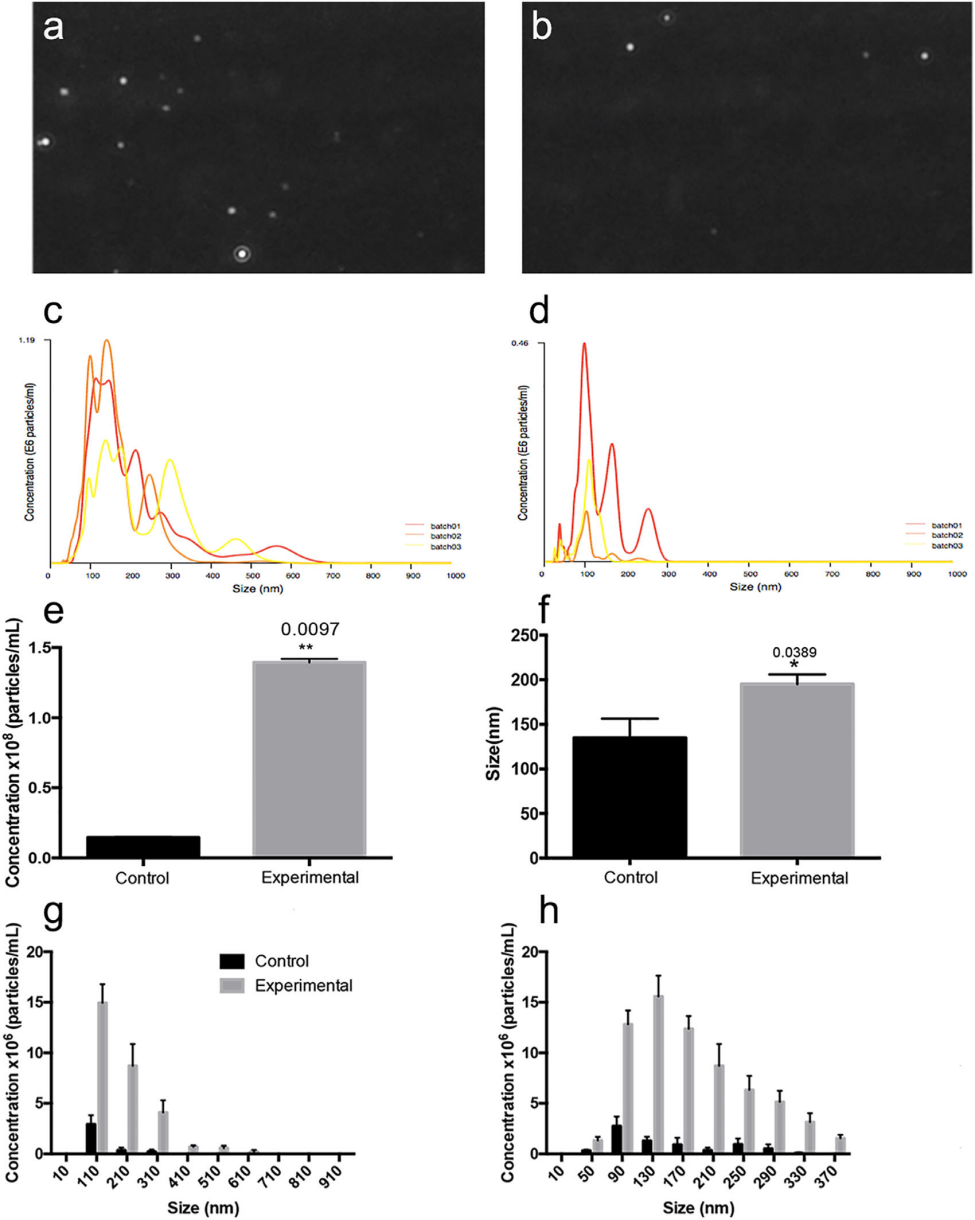
Retinal EV release rate and diameter range. Nanosight laser diffraction analysis of individual EVs in single frames of tracking for (**a**) conditioned media and (**b**) control medium. Concentration and diameter traces from (**c**) retina conditioned media and (**d**) control medium reveal variation in concentration and range of diameters. (**e**) Nanoparticle Tracking Analysis (NTA) revealed significantly more EVs in retina-conditioned medium (1.42 +/− 0.08 × 10^8^/mL) compared to those present in control (0.15 +/− 0.09 × 10^8^; *P* = 0.0097). (**f**) NTA analysis revealed a significant difference in mean diameter of EVs released from adult retina, 202 +/− 21.7 nm, compared to EVs in control medium 120 +/− 9.9 nm (*P* = 0.0389). Retina EVs were most concentrated in diameter ranges from 50 to 610 nm, compared to 50 to 410 nm in controls (**g**). (**h**) At each diameter analyzed from 50 to 370 nm, retina EV concentrations were greater than those present in controls.

The concentration and diameter ranges of EVs released from adult retina in culture were characterized using NanoSight analysis. Still images from NanoSight laser diffraction videos showed greater numbers of EVs suspended in retina-conditioned media (Fig. 1a), compared to control media (Fig. 1b). Raw data traces of retina EVs and controls showed intrinsic sample variations and size distributions (Figs. 1c,d), respectively. Nanoparticle Tracking Analysis (NTA) revealed significantly more EVs in retina conditioned media (1.42 +/− 0.08 × 10^8^/mL) compared to control medium (0.15 +/− 0.09 × 10^8^; *P* = 0.0097; Fig. 1e). These data indicate that, in the defined culture conditions at 37°C, an individual retina releases approximately 1042 EVs per hour. A sample of Brownian motion exhibited by retina EVs in solution at 24 hours is provided in Supplementary Video S1. NTA analysis also revealed a significant difference in mean diameter of EVs released from adult retina (202 +/− 21.7 nm), compared to EVs in control medium (120 +/− 9.9 nm; Fig. 1f; *P* = 0.0389). Retina EVs were concentrated in diameter ranges from 50 to 610 nm, compared to 50 to 410 nm for control medium EVs (Fig. 1g). Lower concentrations of retina EV sizes were detected at 710 nm, 810 nm, and 910 nm at of 0.015 × 10^6^/mL, 0.01067 × 10^6^/mL, and 0.00017 × 10^6^/mL, respectively.

### Transmission Electron Microscopy Immunogold TSG101 and CD63 Labeling of Retinal EVs

**Figure 2.**
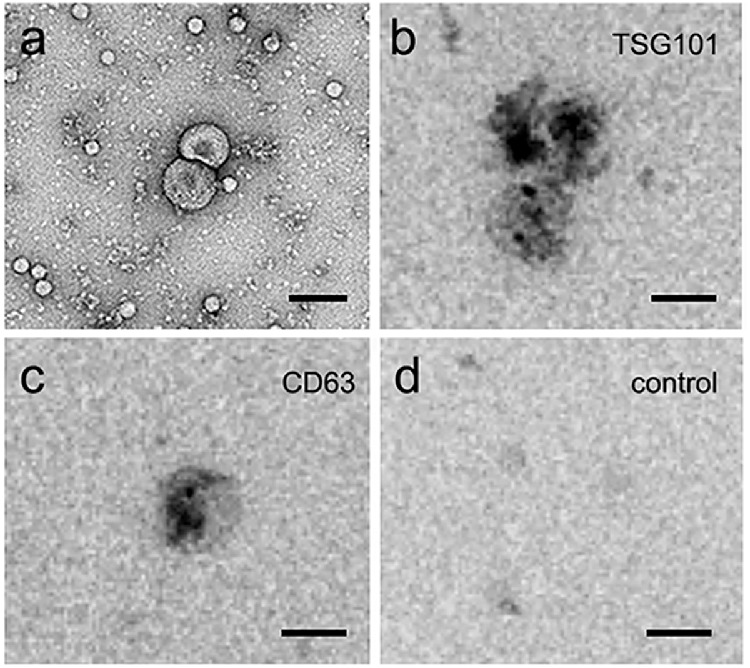
TEM and immunogold EM of retinal EV ultrastructure and protein localization. TEM analysis of EVs released from adult retina revealed cup-shaped and spheroid morphologies, characteristic of exosomes and microvesicles, respectively (Scale: 100 nm). Retinal EVs also showed positive immunogold-labeling with canonical EV markers including (**a**) the MVB synthesis protein, tumor susceptibility gene 101 protein (TSG 101), and (**b**) the transmembrane extracellular protein CD63. In retina EV controls with secondary antibody alone, (**c**) no labeling was detected. Scale: 100 nm.

To further characterize the presence of EVs within retina-conditioned media, isolated EVs were analyzed using transmission electron microscopy (TEM; Fig. 2). TEM revealed spheroid and cup-shaped EV ultrastructures, supporting characterization as exosomes and microvesicles, respectively (Fig. 2a). Two canonical EV markers were used for immunogold TEM, the MVB synthesis protein, tumor susceptibility gene 101 protein (Tsg101; Fig. 2b) and the tetraspan protein CD63 (Fig. 2c). EVs labeled positively for both markers and negative controls, without primary antibody, showed no labeling (Fig. 2d).

### Molecular Content of Retinal EVs

**Figure 3.**
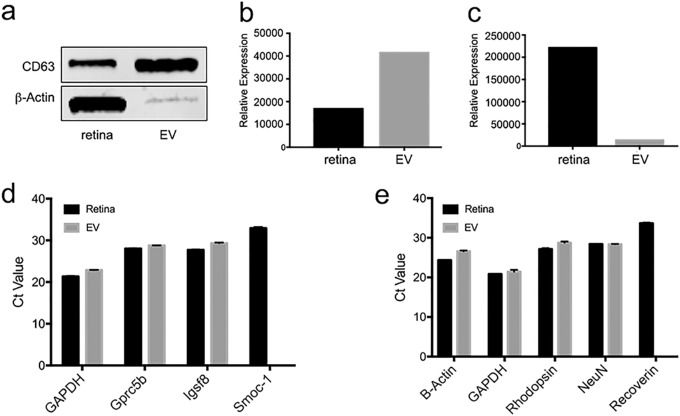
Retinal EV marker protein and select retinal mRNA content. Western blot analysis of 5 µg of protein analyzed from EVs and retina revealed, (**a**) the canonical exosome marker CD63 is concentrated in EV samples, with (**b**) CD63 intensity quantification showing higher levels of CD63 in EVs compared to retina (**c**) beta-actin showed higher levels in retina proteins compared to equal amount of EV proteins. qRT-PCR analysis showed (**d**) retina and EV expression levels of transcripts associated with neural differentiation and organization, including GPRC5b and Igsf8. Smoc1 was found in retina but not detected in EVs. Analysis also showed (**e**) adult retinal and neuronal markers in both retina and EVs, including rhodopsin and Neun. Recoverin was only detected in retinal cells.

The molecular content of retina EVs (RNA and protein) was analyzed and compared to that of the adult retina of origin. As described, TEM results showed co-localization of CD63 to the EV lipid wall. The concentration was validated using Western blot, which revealed that CD63 was at higher levels in EVs relative to retina samples (Figs. 3a−c). Next, total RNA was isolated from retinas and EVs for qRT-PCR analysis. Target genes selected for RNA analysis were identified from recent retinal progenitor cell EV analysis and were associated with retinal organization and function.^30^ The EV total RNA was analyzed for the presence of SPARC-related modular calcium binding 1 (Smoc-1), G protein-coupled receptor 5b (Gpcr5b), and immunoglobulin superfamily member 8 (Igsf8), using glyceraldehyde-3-phosphate dehydrogenase (GAPDH) as control (Fig. 3d). Smoc-1 has been shown to localize to Muller cells during retinal development and functions as a calcium binding protein in pericellular matrices.^31^ Igsf8 is an immunoglobulin protein that binds tetraspanin molecules and regulates synapse structure through CD9 cell adhesion molecules.^32^ Gprc5b is enriched in cone photoreceptors and associated with retinal development and physiology through interaction with retinoic acid signaling pathways.^32^^,^^33^ The initial gene expression analysis revealed that retinal EVs contained Gprc5b and Igsf8 mRNA (Fig. 3d). The expression levels observed in retinas were comparable to those in EVs, with the exception of Smoc1 that was present in retinas but not in EVs.

In addition to analyzing for retinal organization markers present in EVs, qRT-PCR was performed to detect transcripts of established markers of retinal neural identity and function, such as rhodopsin, recoverin, and the RNA Binding Protein Fox-1 Homolog 3 (Neun; Fig. 3e). Analysis revealed the presence of rhodopsin and Neun in EVs, whereas recoverin levels were detected only in the retina.

**Figure 4.**
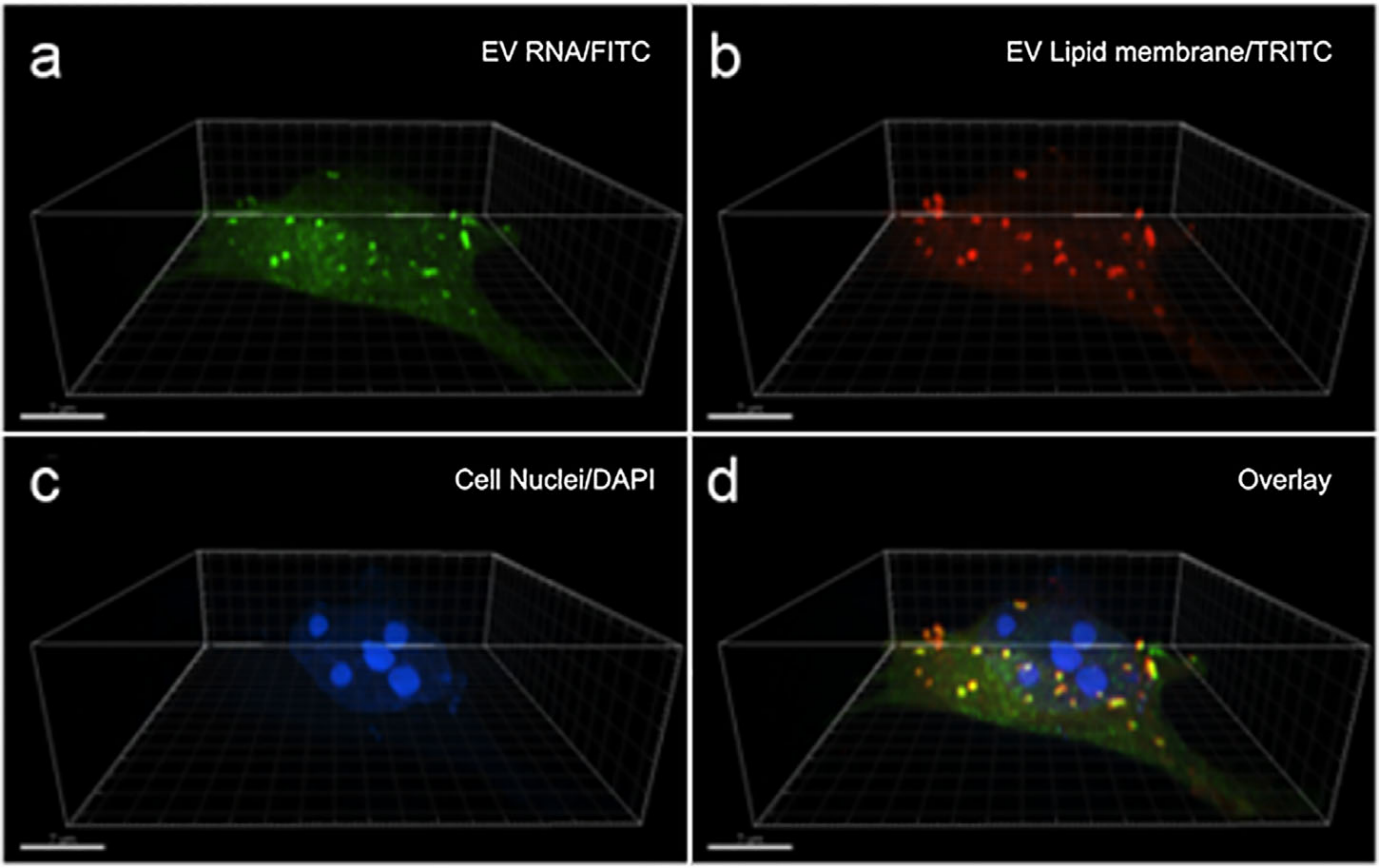
Lipid membrane and RNA labeled EVs released from adult retina are up-taken by unlabeled retinal cells. Super-resolution microscopy was used to visualize RNA (SytoRNA/green) and lipid membrane (PKH26/red) labeled EVs released from adult retina and taken up by control non-labeled adult retinal cells in a transfer experiment. Shown are adult retinal cells with internalized EVs imaged for transferred (**a**) total RNA (FITC/green) and (**b**) lipid membrane (TRITC/red) with (**c**) nuclei labeled with (DAPI). Overlay revealed, (**d**) colocalization of transferred EVs with visible RNA and lipid membrane throughout the cytoplasm and in the proximity of the perinuclear envelope of the receiving retinal cells.

To visualize potential genetic EV cargo transfer in the adult retinal microenvironment, we imaged EVs released from adult retina with labeled total RNA (SytoRNA/green) and lipid membrane (PKH26/red). Labeled adult retina released FITC-detected RNA cargo within TRITC-detected lipid-enclosed EVs through transwell pores, and the uptake of these EVs was visualized in nonlabeled adult retinal cells in the lower wells using super-resolution microscopy (Fig. 4). Following 5 days of transfer, labeled EVs were observed localized within the cytoplasm, often close to endoplasmic reticulum and nuclear membrane, within the receiving adult retinal cells.

### Proteomic Analysis of Retina and EV Cargo

**Figure 5.**
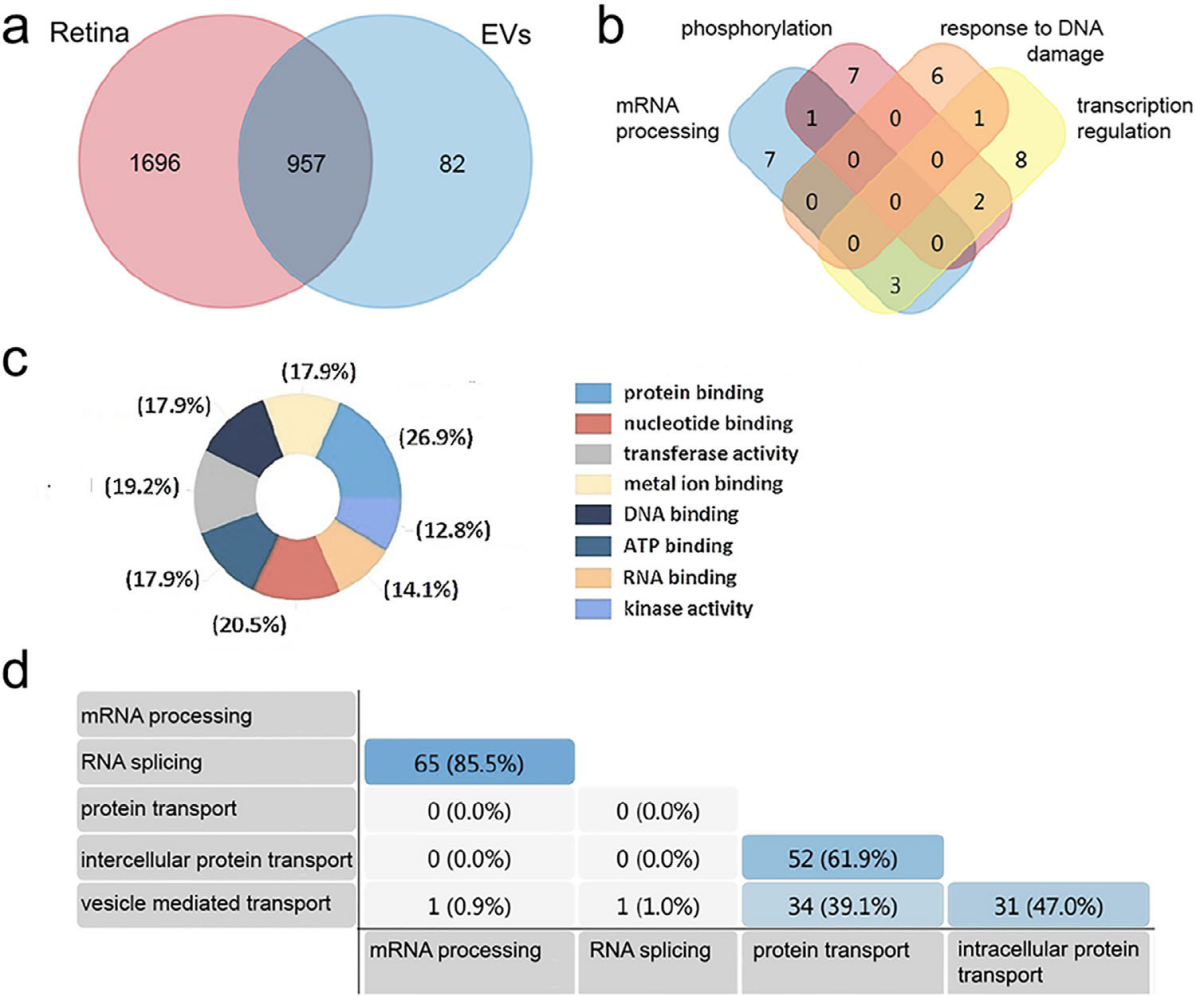
Retinal EVs contain unique protein profiles compared to retina. A Venn diagram showing, (**a**) the distribution of the 2732 proteins detected in retina, EVs or both. Of the proteins identified, 957 were present in both retina and EVs, 1696 were detected only in retina, and 82 in EVs alone. (**b**) Venn diagram showing the top biological processes associated with the 82 proteins detected in EVs alone. (**c**) The percent distribution of molecular processes associated with proteins identified only in EVs. (**d**) The table shows the percent distribution of the biologic processes associated with total EV protein cargo, including protein species detected exclusively in EVs (82) and EV cargo shared with retina (957).

LC-MS analysis was performed on retinal tissues and EVs to characterize protein species found in both samples. Proteomic analysis identified a total of 2735 proteins; of these, 1696 were in retina alone, 957 in both retina and EVs, and 82 detected in EVs alone (Fig. 5A). Supplemental Tables S1a, S1b, and S1c list proteins exclusively detected in retina, in EVs shared between retina and EVs and detected exclusively in EVs, respectively.

Bioinformatic analysis was then performed to model EV protein function in adult retinal microenvironments. The bioinformatics network mapping software FUNRICH was utilized to analyze EV protein molecular function, biological processes, and molecular pathways.^34^ Within the set of proteins identified in EVs, the top predicted biological processes included mRNA processing, phosphorylation, DNA dependent regulation of transcription, stimulus, and response to DNA damage (Fig. 5B). Supplemental Table S2 lists EV proteins grouped by predicted biologic process as depicted in Figure 5B. The top percentile molecular functions included gene regulation and phosphorylation. The functional categories associated with the EV proteins, by percentage, included protein binding (26.9%), nucleotide binding (20.5%), transferase activity (19.2%), ATP binding (17.9%), DNA binding (17.9%), metal ion binding (17.9%), RNA binding (14.1%), and kinase activity (12.8%; Fig. 5C). Next, EV proteins alone and those shared with cells were correlated to biologic processes, revealing 65 proteins involved with RNA splicing/mRNA processing (85.5%), 52 with intracellular protein transport/protein transport (61.9%), 34 with vesicle mediated transport/protein transport (39.1%), and 31 with vesicle mediated transport/ intracellular protein transport (47.0%; Fig. 5D).

## Discussion

In this work, the characterization of adult retinal EV release, RNA transfer, and proteomic cargo are described. EVs released from normal adult retina appeared heterogeneous in size and morphology, with diameters comparable to EVs studied in other neural cell types.^24^ The full range of retinal EV diameters observed (30−910 nm) suggests release of microvesicles and exosomes potentially from multiple cell types with unique molecular cargo and sizes.^35^ Characterization of retinal EV diameters, concentrations, and molecular cargo may contribute to their development as prognostic biomarkers for retinal disease.^36^^,^^37^

The transfer of EV RNA in this study in vitro suggests that retinal EV cargo transfer may occur in vivo with potential roles in retinal function and pathologic processes. A growing number of studies are reporting the release EVs with cargo involved in CNS homeostasis and disease.^35^ Recent analysis of EVs from RPE describe autophagy conjugate Atg12-Atg5 and other autophagy pathway cargo with predicted roles in cellular homeostasis.^38^ EVs from RPE have also been reported to contain αβ crystalline, mediating anti-inflammatory processes and providing neuroprotection.^39^ Cortical neurons have been observed to release AMPA receptors in exosomes during heightened excitability, with a predicted role in synaptic remodeling.^40^ CNS EVs have been shown to contribute to the pathogenesis of Alzheimer's disease, Parkinson's disease, and multiple sclerosis.^41^

Of the proteins detected exclusively in retinal EVs, a small percentage have predicted roles in retinal structure and function, including the cadherin related family member (Cdhr1), castor zinc finger 1 (Casz1), syndecan-binding protein (Sdcbp), and retinol dehydrogenase 5 (Rdh5). Cdhr1 is an adhesion protein that localizes between the outer and inner segments of photoreceptors and critical for retinal structure and photoreceptor survival.^42^ Casz1 is a zinc finger transcription factor that is predicted to control rod nuclear organization and maintain the rod photoreceptor genome.^43^ Casz1 is also involved in retinal progenitor temporal progression and is present in postmitotic cones and certain GABAergic amacrine cells.^44^ Sdcbp is a multifunctional protein with involvement in membrane trafficking, cell adhesion, and neuronal-synapse function.^45^ In the retina, Sdcbp is critical to vision where it binds directly to leucine rich repeat, Ig-like, and transmembrane domain 1 (Lrit1) protein regulating cone transduction.^46^

To begin to determine the cellular origins of adult retinal EVs, we analyzed for gene products in EVs having established expression in photoreceptors, amacrine, and ganglion cells. Rod photoreceptors exhibit cell specific expression of rhodopsin, which was identified in isolated adult retinal EVs.^47^ Rhodopsin is the photo-responsive G-coupled receptor of rod cells. There are approximately 6.4 million rod cells in each mouse retina.^48^ In addition, transcripts for the neuronal-specific nuclear protein (Neun), a marker for subpopulations of amacrine and ganglion cells, was present in retinal EVs.^49^ Neun is a nuclear protein predicted to function as an RNA binding protein involved in regulation of splicing.^50^ The presence of cell-specific molecular cargo in adult retinal EVs may indicate release from photoreceptors, horizontal cells, or ganglion cells. Further analysis will help elucidate the EV profiles and function of individual retinal cell types.

In conclusion, this study provides an initial characterization of adult neural retina EV release rate, as well as EV encapsulation of RNA and protein. Adult retina constitutively releases EVs with mean diameters of 202 +/− 21.7 nm. The data show that labeled adult retinal EVs and their RNA cargo transfer to adult retinal neurons and are internalized near the endoplasmic reticulum and nucleus. Retina EV mRNA and proteome analysis reveals that EV cargo have strongly predicted associations with phototransduction, synapse structure, RNA processing, and transcription regulation.

## Supplementary Material

Supplement 1

Supplement 2

## Supplementary Material

**Supplementary Video**. Nanosight imaging of adult retina EVs released into culture media at 24 hours reveals movement patterns associated with Brownian motion and diffusion. Analysis of this data using NTA determines EV concentration and diameter.
